# Management of a rare fixed knee flexion deformity in adulthood following childhood osteoarticular infection: A case report and literature review

**DOI:** 10.1097/MD.0000000000044283

**Published:** 2025-09-12

**Authors:** Ji Wu, Sihe Qin

**Affiliations:** a Department of Pediatric Orthopedics, Wuhan Children’s Hospital (Wuhan Maternal and Child Healthcare Hospital), Tongji Medical College, Huazhong University of Science & Technology, Wuhan, China; b Department of Orthopedics, Rehabilitation Hospital Affiliated to National Rehabilitation Aids Research Center, Beijing, China.

**Keywords:** fixed knee flexion deformity, Ilizarov technique, limb shortening, osteoarticular infection

## Abstract

**Rationale:**

Severe fixed knee flexion deformities and significant limb shortening resulting from childhood osteoarticular infections are uncommon in clinical adult orthopedics and present considerable surgical challenges.

**Patient concerns:**

A 23-year-old female presented with a 130° fixed knee flexion deformity and severe limb shortening. Despite recommendations for amputation from multiple hospitals, the patient insisted on pursuing limb-preserving surgical options.

**Diagnoses:**

The patient was diagnosed with a severe fixed knee flexion deformity and significant limb shortening due to childhood pyogenic knee infection.

**Interventions:**

A staged surgical strategy was adopted. The first stage included wedge osteotomy combined with the Ilizarov technique for deformity correction and partial limb length restoration. The second stage utilized femoral and tibial osteotomies with the Ilizarov method for further lengthening, complemented by steel pin stimulation for delayed tibial healing.

**Outcomes:**

At the 7.5-year follow-up, the patient achieved near-equal limb lengths, restored walking ability, and substantial functional improvement. At the 11-year follow-up, the patient retained the ability to walk independently. Residual knee joint stiffness was observed but did not significantly impact daily activities.

**Lessons:**

This case demonstrates the effectiveness of the Ilizarov technique in correcting extreme deformities and achieving significant limb lengthening. Its success highlights the importance of systematic preoperative evaluation, meticulous surgical planning, and strong patient collaboration. Furthermore, this case highlights the critical need for long-term monitoring of pediatric osteoarticular infections to mitigate the risk of subsequent limb deformities during growth and development.

## 1. Introduction

Pediatric osteoarticular infections, such as pyogenic knee arthritis, can lead to irreversible damage to the bone and joint structures if not treated promptly and effectively. As the child grows, this damage can result in severe functional impairment and deformity in adulthood.^[1,2]^ These sequelae not only severely impact the patient’s physiological functions but can also have profound impacts on their social and psychological well-being. Even with appropriate diagnosis and treatment, these infections can still pose significant risks to the patient’s life or limbs.^[3]^ To the best of our knowledge, there is limited literature on severe knee flexion deformities and limb shortening resulting from childhood osteoarticular infections in adulthood. In this study, we present an unusual case of severe osseous knee flexion deformity and limb shortening as a sequela of pediatric osteoarticular infection, which significantly improved after treatment. Through a 2-stage surgical approach spanning more than 3 years, we successfully reconstructed limb function and restored the morphology of this atypical and complex deformity using the Ilizarov technique. This case underscores the potential of the Ilizarov technique in managing unusual and complex orthopedic conditions; furthermore, it highlights the critical importance of a comprehensive and individualized treatment strategy in such rare and challenging cases.

## 2. Case presentation

### 2.1. Patient information

#### 2.1.1. Medical history

**Figure 1.**
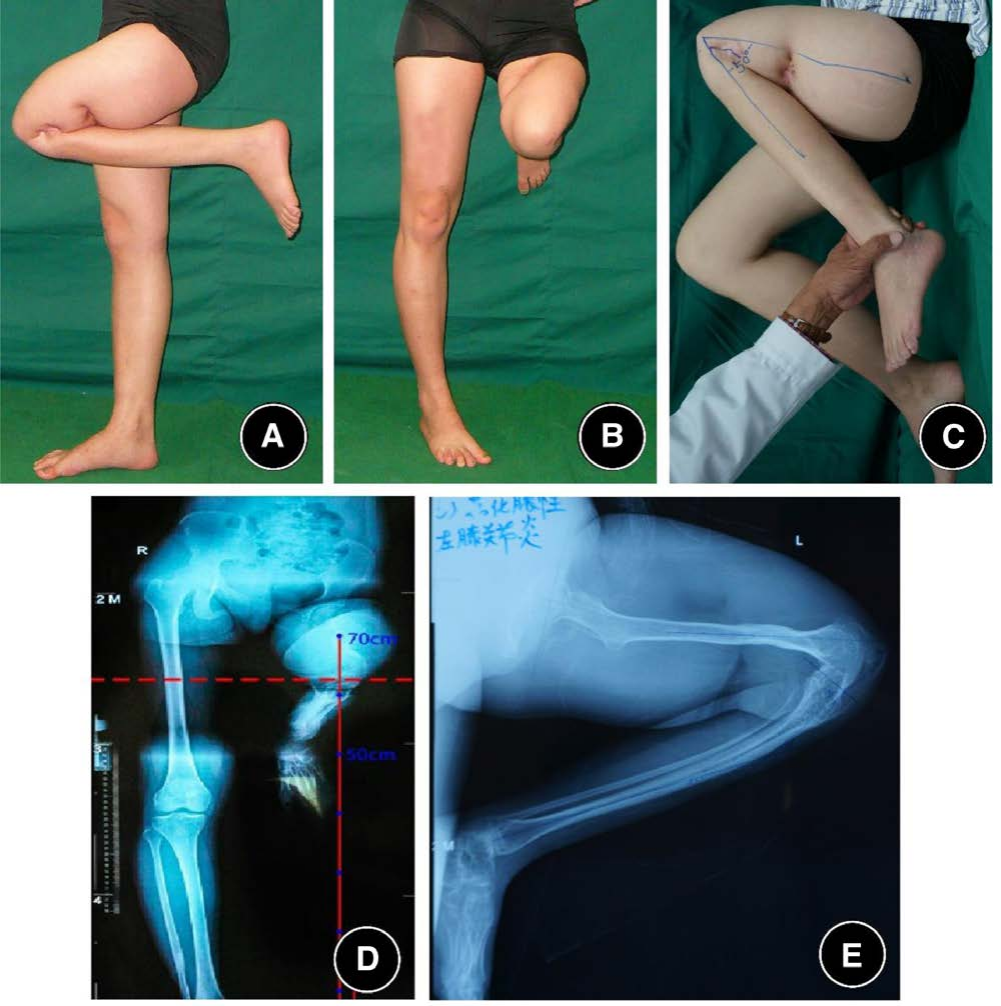
Preoperative examination showing a 130° knee flexion deformity, with scarring adhesions between the thigh and calf. (A) Clinical photograph showing a fixed ~130° knee flexion deformity. (B) Standing photograph illustrating that the left knee remains >60 cm above the floor due to the fixed flexion. (C) Close-up of the popliteal region demonstrating extensive scarring and adhesions between the posterior thigh and proximal leg.(D) Anteroposterior radiograph illustrating that the left knee remains >60 cm above the floor due to the fixed flexion. (E) Lateral radiograph confirming osseous ankylosis of the knee in ~130° of flexion, with gracile femur and tibia; the bony structures of the hip and ankle appear preserved.

This case involves a 23-year-old female. At the age of 5, the patient suffered from a pyogenic infection in the left femur and knee joint. After over 2 years of repeated treatments, the infection was eventually cured. However, the knee joint sustained significant damage, resulting in osseous ankylosis in a flexed position. As the patient grew, the knee flexion deformity progressively worsened. Due to the lack of corrective treatment during adolescence, the patient is currently only able to walk with the assistance of a cane, relying solely on the right lower limb. The patient sought consultation at many hospitals across China, all of which recommended amputation and prosthetic fitting. However, she was resolute in her decision to preserve her limb and sought further evaluation in October 2013 at the orthopedic department of Sihe Qin in Beijing. Upon initial examination, the knee flexion deformity was 130 degrees, with significant scarring and adhesions between the thigh and lower leg (Fig. 1).

#### 2.1.2. Physical examination

The patient was in good general health and displayed an active personality. The left knee joint exhibited a fixed flexion deformity of 130° (Fig. 1), with significant skin scarring at the affected joint and approximately 15° of hip flexion contracture. When standing, her knee was positioned more than 60 cm from the floor. She utilized bilateral crutches and depended on her right lower limb for ambulation and weight-bearing.

The range of motion in the knee joint was severely restricted, with the knee fixed in a 130° flexion position. According to the knee society scoreKnee Society Score (KSS) system,^[4]^ the motion score was 0 points. Functionally, the patient was highly dependent on bilateral crutches and entirely relied on the right lower limb for weight-bearing. The corresponding knee society score functional score for her left knee was nearly 0 points.

#### 2.1.3. X-ray examination

X-ray imaging revealed that the knee joint was fused in a flexed position, with a flexion angle exceeding 130°. The femur and tibia appeared notably slender, whereas the bony structures of the hip and ankle joints were mostly normal (Fig. 1).

### 2.2. Case analysis and surgical reconstruction strategy

#### 2.2.1. Clear indications for deformity correction and functional reconstruction surgery

In accordance with the patient’s request for treatment of the left lower limb, the treatment objectives included preserving the limb, achieving basic knee extension at the osseously ankylosed knee joint, ensuring near-equal limb length between both lower limbs, and enabling weight-bearing and walking on both legs.

#### 2.2.2. Staged surgical approach

Considering the patient’s specific needs, along with the clinical expertise and technical capabilities of the Orthopedic Department of Sihe Qin, it was anticipated that the treatment goals could be achieved through a staged surgical approach involving 2 or more procedures. The basic reconstruction strategy was outlined as follows: a first-stage surgery to correct the knee flexion deformity and significantly restore lower limb length, allowing the patient to walk with weight-bearing on both legs; a second-stage surgery to perform limb lengthening to achieve equal limb lengths in both lower limbs.

#### 2.2.3. Challenges to overcome and risk mitigation for both the surgeon and patient

Given the knee flexion deformity exceeding 130° and the presence of scar contracture at the posterior knee, along with the slender femur and tibia, the risk of delayed bone healing during limb lengthening was considerable. The surgical objectives were to achieve knee extension and approximate equal limb length, with the expectation that more than 2 surgeries would be required to regenerate over 30 cm of length. The total treatment and recovery period could extend beyond 2 years. Despite these challenges, the patient remained highly motivated to regain the ability to walk on both lower limbs and showed a high level of cooperation throughout the treatment process.

### 2.3. First-stage surgery: wedge osteotomy of the knee combined with Ilizarov technique for correction of knee flexion deformity

#### 2.3.1. Basic surgical steps

The first surgery was performed in October 2013. During the procedure, a wedge shortening osteotomy of the distal femur was performed to correct the fixed flexion deformity of the left knee joint. Surrounding adhesions and scar tissue were carefully released, resulting in an immediate correction of approximately 40° of knee flexion deformity. After suturing the incision, pins were inserted, and an Ilizarov external fixator was applied. Two Ilizarov rings were installed on both the femur and tibia for further correction postoperatively. Upon awakening from anesthesia, the patient was examined, and no signs of traction-induced paralysis of the common peroneal or tibial nerve were observed. Circulation in the lower leg was normal, and the patient was subsequently returned to the ward. Following Paley principles of osteotomy and deformity correction, a framework for the external fixator was constructed with the aim of resolving the knee flexion deformity and adjacent deformities of the CORA (center of rotation of angulation) through open wedge osteotomies and translations. During the lengthening phase, the hinge was set to the distraction mode.

#### 2.3.2. Postoperative management

**Figure 2.**
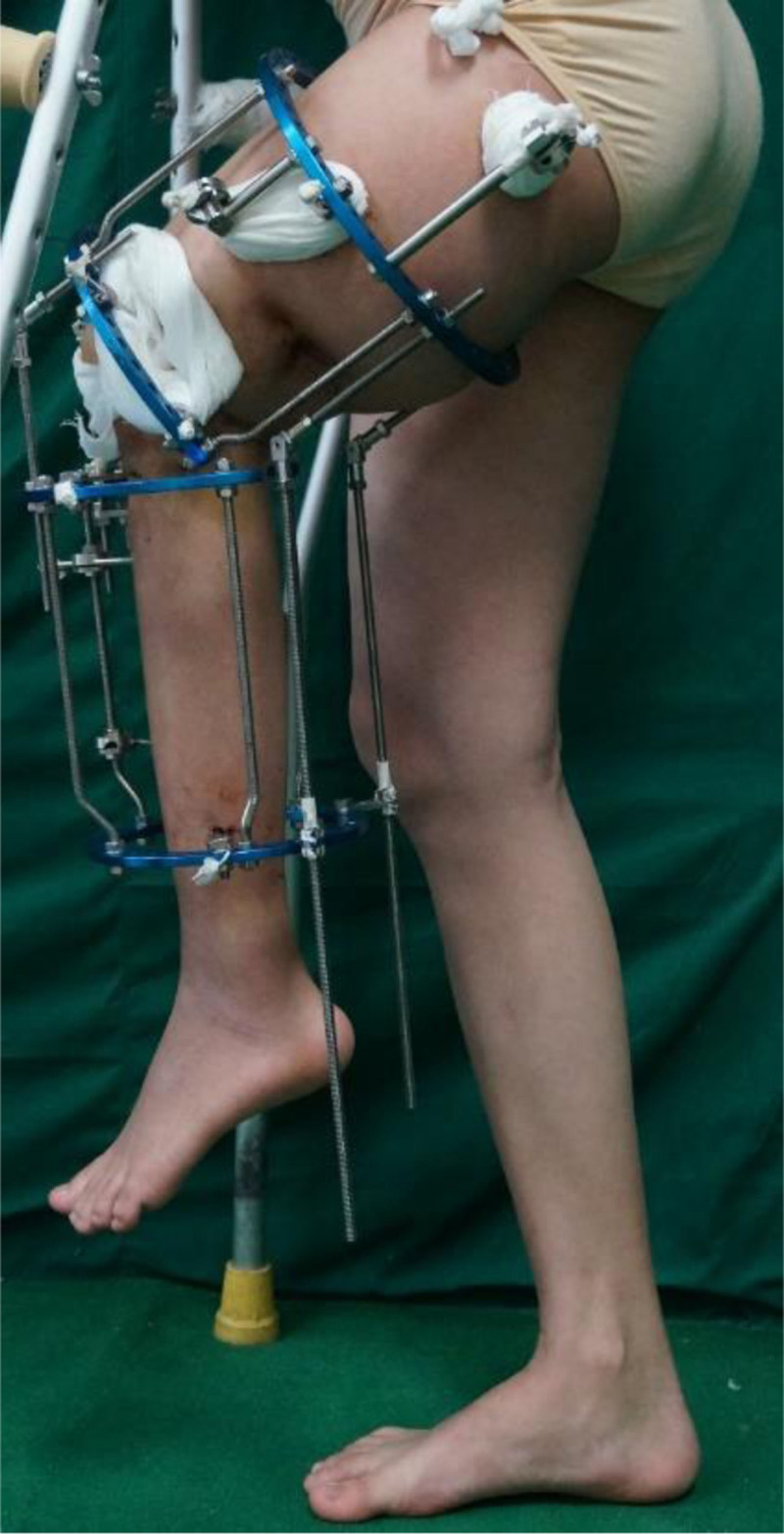
Postoperative appearance on the 5th day following surgery.

**Figure 3.**
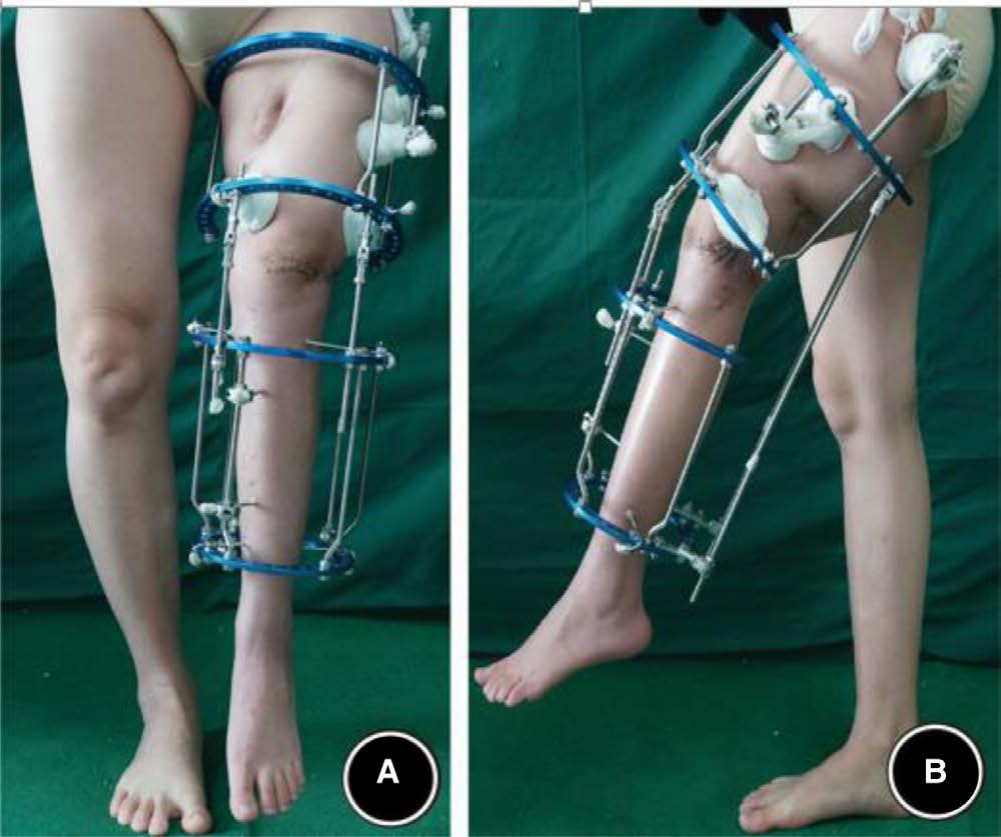
Postoperative appearance on the 84th day, with significant improvement in the knee flexion deformity, enabling weight-bearing walking with the use of customized shoe insoles. (A) Frontal view clinical photograph. (B) Lateral view clinical photograph.

**Figure 4.**
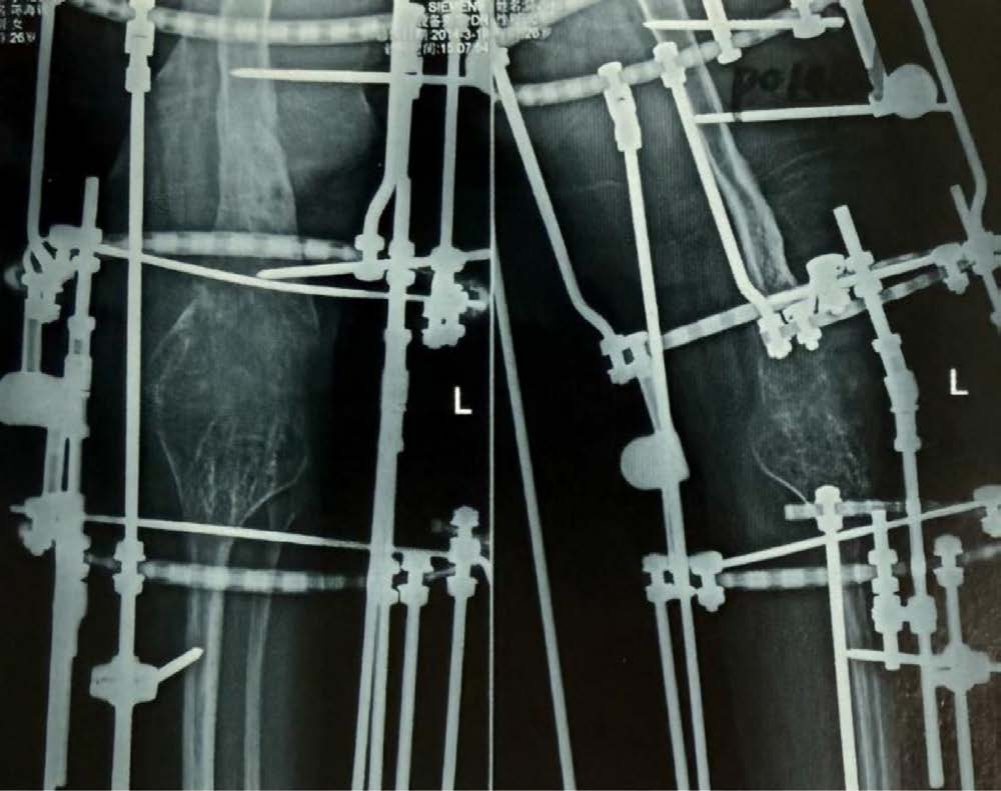
X-ray examination at 128 days postsurgery showing good bone healing at the knee osteotomy site.

On the fifth day postsurgery, X-ray images confirmed the correction effect, and gradual traction was initiated to correct the remaining knee flexion deformity. The patient and her family were instructed on how to adjust the distraction rods, allowing flexibility in controlling the speed of traction based on the patient’s subjective sensations (Fig. 2). By the 84th postoperative day, the knee flexion deformity was nearly completely corrected, and weight-bearing walking could be achieved using custom-made shoe lifts (Fig. 3). X-ray examination on postoperative day 128 showed good bone healing at the osteotomy site (Fig. 4). Four months after the first surgery, the patient was re-evaluated. The knee flexion deformity was largely corrected, and the patient was able to walk with weight-bearing on both lower limbs. She was extremely pleased to experience weight-bearing ambulation with both legs. Although the left lower limb had been lengthened by more than 24 cm, a residual 16 cm shortening remained. The patient requested further lengthening surgery to restore the length of the left lower limb.

### 2.4. Second-stage surgery: bilateral femoral and tibial osteotomy and lengthening

**Figure 5.**
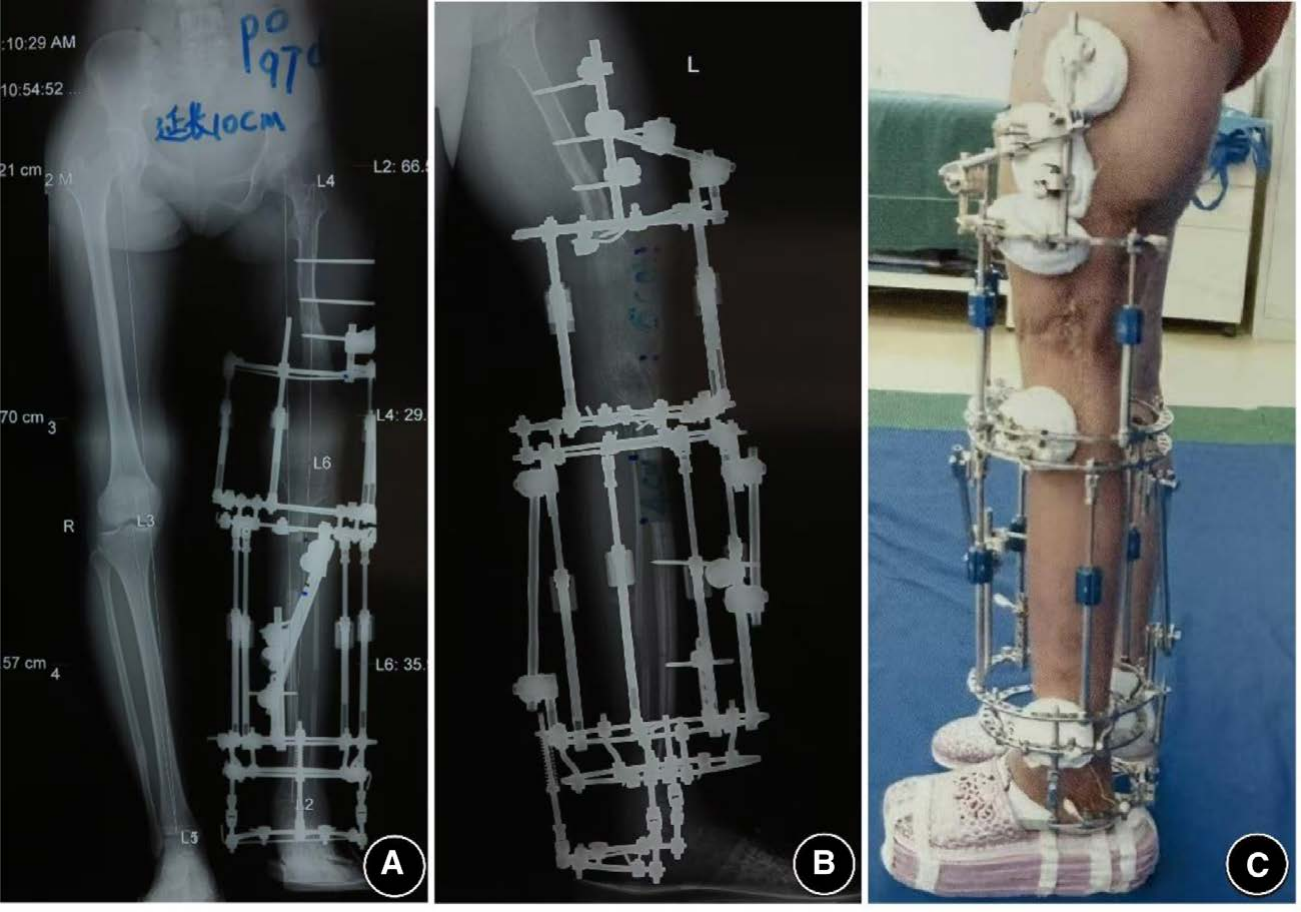
Postoperative day 97 following the second surgery, showing approximately 10 cm of limb lengthening (4 cm tibial lengthening and 6 cm femoral lengthening). (A) Standing anteroposterior radiograph of both lower limbs on postoperative day 97, demonstrating an Ilizarov circular external fixator with bifocal lengthening at the distal femur and proximal tibia of the left side. (B) Standing lateral radiograph of the left lower limb demonstrating ~6 cm femoral lengthening at the distal femoral osteotomy and ~4 cm tibial lengthening at the proximal tibial osteotomy, with columnar regenerate formation at both sites.(C) Lateral weight-bearing clinical photograph of the left lower limb.

After the correction of the left lower limb flexion deformity, a residual shortening of 16 cm remained in the left lower limb, necessitating an additional lengthening of at least 10 cm to achieve balanced bilateral lower limb length. A bilateral femoral and tibial osteotomy lengthening procedure was performed, involving transverse osteotomies at the distal femur and proximal tibia, followed by application of the Ilizarov circular external fixator (Fig. 5).

**Figure 6.**
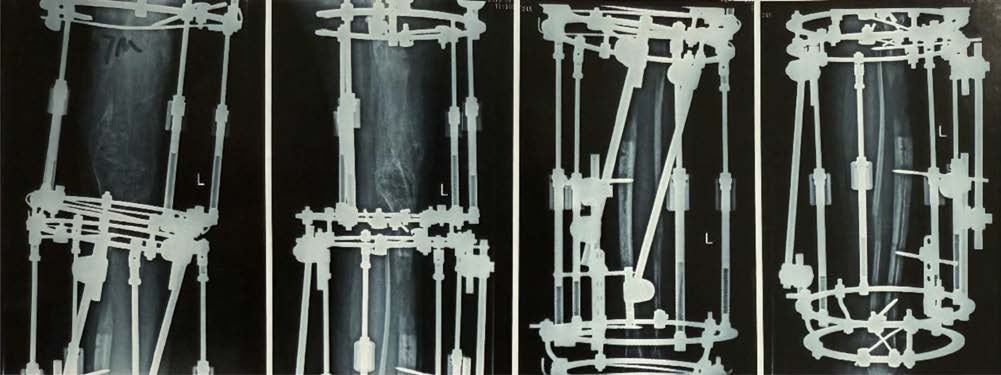
After a 12 cm limb lengthening, the lengthening process was stopped, and 7 months postoperatively, follow-up imaging shows poor bone healing at the tibial lengthening site.

**Figure 7.**
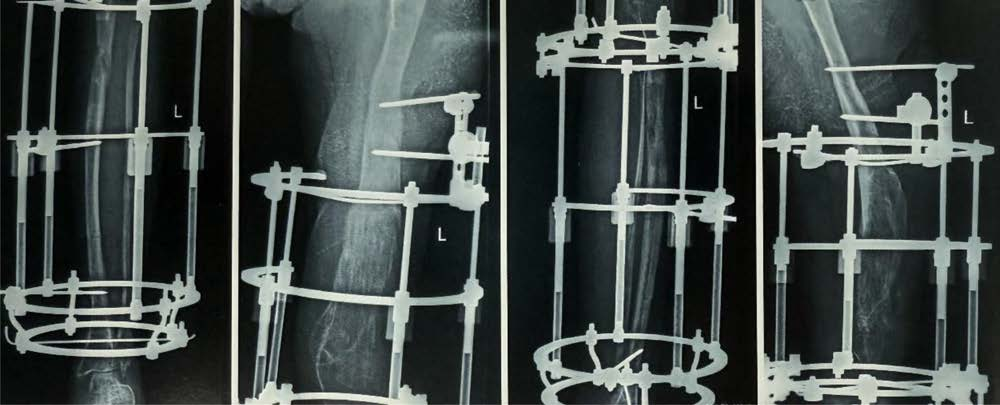
Follow-up at 23 months postoperative, showing inadequate bone healing in the tibial lengthening region, with a percutaneous steel pin stimulation procedure performed under anesthesia to promote osteogenesis.

Postoperatively, the 2 osteotomies were lengthened synchronously at a controlled pace, achieving an overall lengthening of 12 cm. However, during this process, bone healing at the tibial lengthening site was delayed (Fig. 6). To address this, a steel pin stimulation procedure was performed 23 months postsurgery to accelerate bone healing at the tibial site (Fig. 7).

### 2.5. Follow-up results

#### 2.5.1. Three years after the first surgery

**Figure 8.**
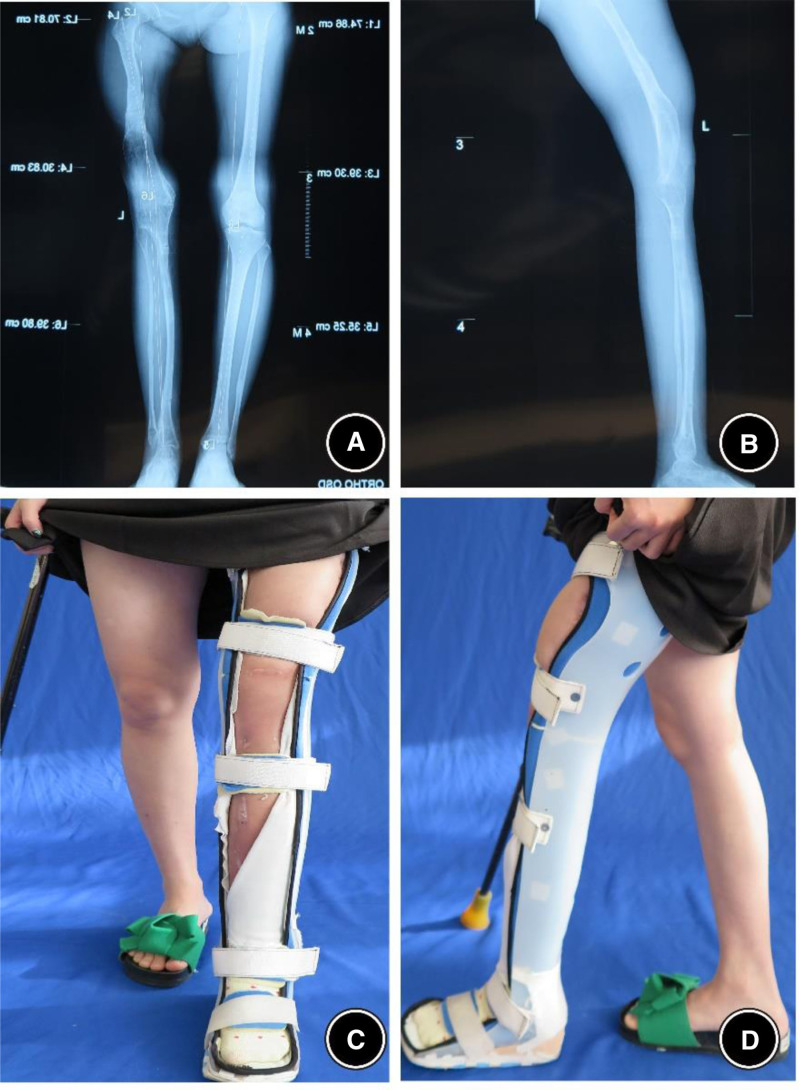
Three years postoperatively, showing significant correction of the fixed knee flexion deformity, with good bone healing in both femoral and tibial lengthening sites, and near-equal length of both lower limbs. (A) Standing anteroposterior radiograph showing near-equal limb. Length and consolidated regenerate at the femoral lengthening site. (B) Standing lateral radiograph. (C) Anteroposterior clinical photograph of the affected limb in standing. (D) Lateral clinical photograph of the affected limb in standing.

The patient returned for a follow-up visit, demonstrating significant correction of the knee flexion deformity. Bone healing at the femoral lengthening site was satisfactory, and the lower limbs were nearly of equal length. The treatment goal of achieving weight-bearing ambulation on both legs was successfully accomplished, with no complications impacting the outcome (Fig. 8).

#### 2.5.2. Seven and a half years after the first surgery

**Figure 9.**
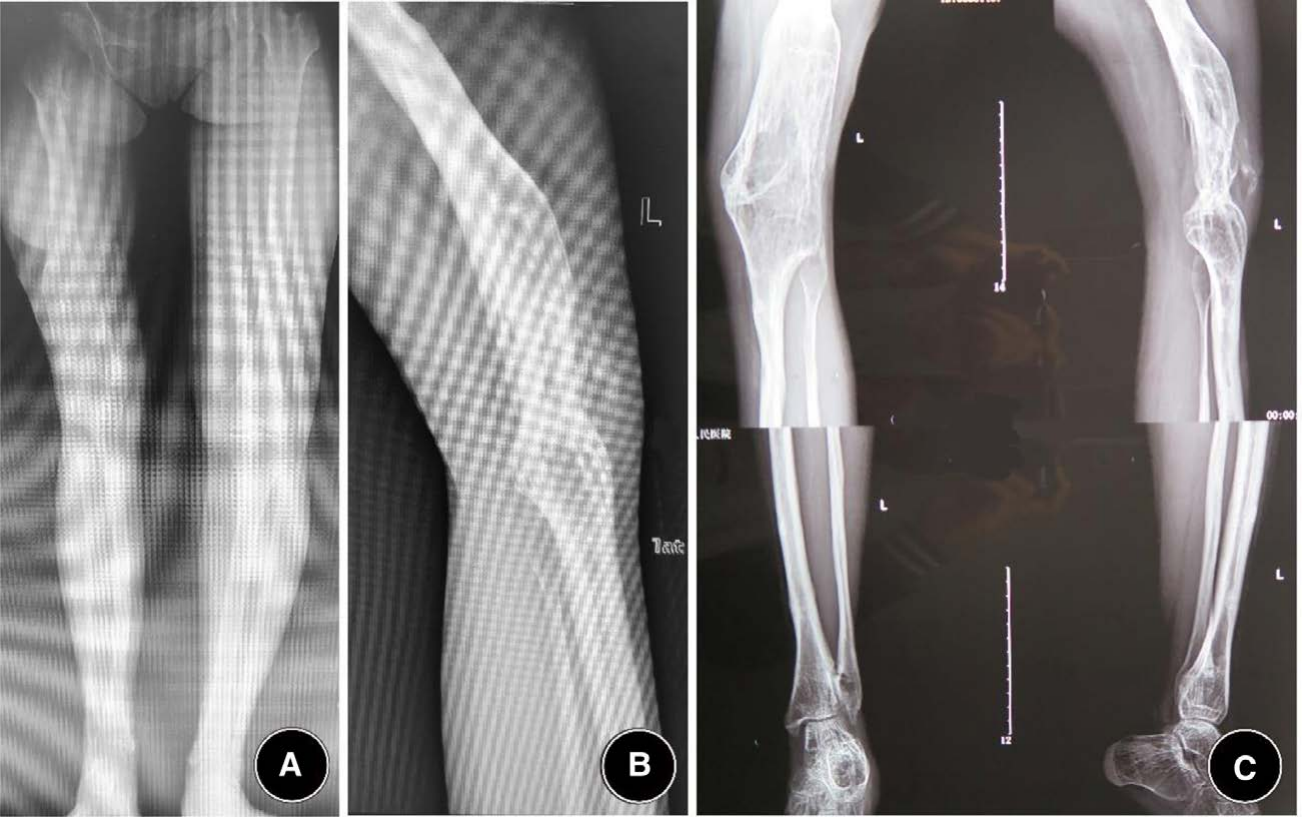
X-ray at 7.5 years postsurgery showing significant improvements in bone density and femoral/tibial diameter. (A) Standing anteroposterior radiograph showing increased trabecular density and cortical thickening of the femur and tibia. (B) Standing lateral radiograph confirming remodeling with increased cross-sectional diameters of the femur and tibia. (C) Magnified anteroposterior view.

X-ray imaging revealed substantial improvements in bone density and the cross-sectional diameters of the femur and tibia (Fig. 9). The lower limbs showed significant increase in circumference, and the patient was able to walk more than 5 kilometers without assistance.

#### 2.5.3. Eleven years after the first surgery, at age 34

**Figure 10.**
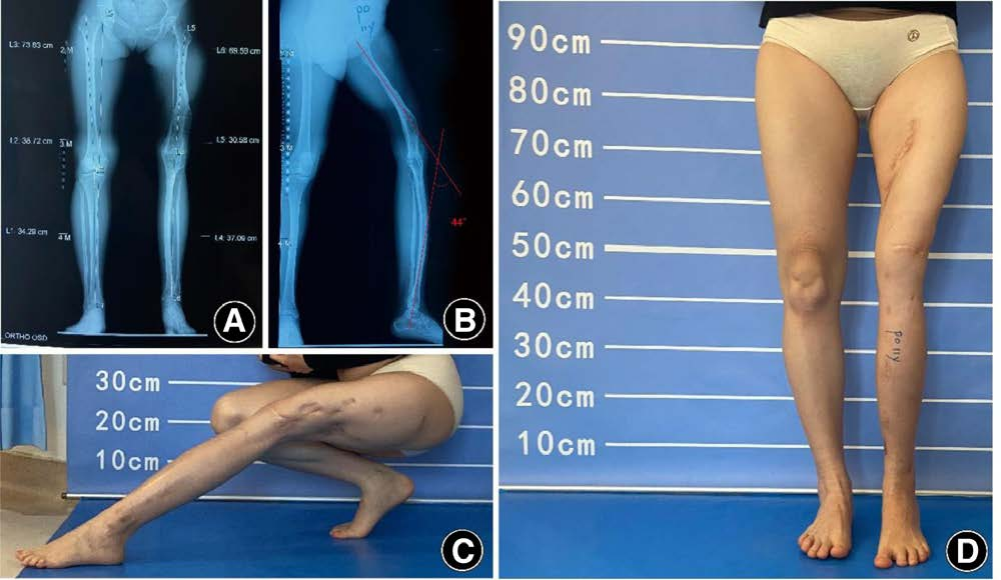
Eleven years postsurgery, at age 34, with left knee bony ankylosis, but able to walk for long durations. (A) Standing anteroposterior radiograph. (B) Standing lateral radiograph. (C) Lateral clinical photograph. (D) Frontal clinical photograph of the lower limbs in standing, demonstrating stable bilateral weight-bearing with visible left quadriceps atrophy and anterior skin rigidity.

The patient showed knee stiffness, with bone ankylosis at the left knee. Although she was able to walk independently for extended periods, quadriceps atrophy and skin rigidity were observed. Consultation with arthroplasty specialists concluded that total knee arthroplasty (TKA) at this stage would likely result in a knee flexion of no more than 60°. It was recommended that she consider knee replacement surgery at the age of 40 (Fig. 10).

## 3. Discussion

Although there are reports in the literature on the treatment of sequelae from pediatric joint infections,^[5,6]^ there is a significant lack of studies on the management of complex adult deformities resulting from childhood joint infections. Such cases are extremely rare, especially those treated with Ilizarov technology. This study not only describes a staged Ilizarov surgical approach but also highlights the benefits of this technique in managing severe deformities, a topic rarely addressed in the literature.

For pediatric fixed knee flexion deformities, less invasive methods with smaller implants are often preferred, as they may help mitigate many complex problems in adulthood. Gaurav et al^[7]^ suggested that for younger children, knee flexion deformities of 15° to 20° or less may be treated with posterior hamstring lengthening and joint capsule release. For severe knee flexion deformities in growing children, treatment may involve iliotibial band and hamstring tendon release, in combination with posterior joint capsule release. Growth modulation for lower limb angular deformities has become a fundamental approach in pediatric orthopedics.^[8,9]^ Herzenberg recommended the use of bilateral anterior tension band plates, rather than paired screws, for sagittal plane growth modulation in the treatment of pediatric knee flexion deformities.^[10]^ The use of tension band plates for growth modulation is a safe and effective method for correcting various deformities in skeletally immature patients, whether idiopathic or pathological.^[11]^

If a knee joint infection in childhood is not treated promptly and effectively, it can lead to irreversible joint damage and functional impairment, ultimately resulting in deformities that are difficult to correct, such as severe knee flexion contractures.^[1,2]^ In managing severe lower limb deformities in adults due to childhood bone and joint infections, clinicians often face significant challenges. These challenges stem from both the complexity of treatment and surgical techniques, as well as the rarity of such cases in adults, hindering the accumulation of experience and the establishment of standardized treatment strategies. Our study provides a comprehensive report on the treatment of complex deformities in adulthood, a topic relatively scarce in previous research, emphasizing the importance of early intervention and the potential for corrective treatment in later years through Ilizarov techniques. Timely intervention can reduce the spread and severity of infection, preventing further deformity progression. For these patients, more complex and high-risk surgical interventions may be required in adulthood.

There are several surgical approaches for the treatment of severe knee flexion contractures in adults, including TKA, amputation, and Ilizarov techniques. TKA is typically indicated for severe joint destruction and functional disability; however, in younger patients, the durability of prostheses and the potential need for multiple revisions make it a suboptimal choice. Moreover, TKA does not provide limb lengthening, making it unsuitable for cases that require significant limb lengthening correction. Even after TKA, fixed knee contractures may persist. Knee stiffness is a well-known complication following TKA.^[12]^ Patients with post-TKA stiffness may experience residual pain and functional impairment in the knee joint.^[13,14]^ Agarwal et al^[15]^ reported on 32 adult patients who underwent TKA due to fixed flexion deformity, showing minimal improvement in clinical knee scores. Kim et al^[12]^ studied 39 patients who underwent revision surgery due to post-TKA knee stiffness, concluding that even with revision, the improvement in range of motion was partial, without significant improvement in functional scores, and 25.6% of patients required a second revision. Amputation should be considered only as a last resort when other methods fail to correct the deformity. While walking ability can be restored with a prosthesis after amputation, the psychological and functional impacts of losing a natural limb present lifelong challenges that patients must face. Schober et al^[16]^ found amputation to be paradoxical for many individuals. Many patients experience significant emotional and mental challenges postamputation, feeling deeply lost and saddened. Additionally, patients express concerns about their new lifestyle, particularly regarding the financial implications.

The advantage of Ilizarov technology lies in its potential for bone and soft tissue regeneration, especially in cases requiring substantial limb lengthening or complex 3-dimensional deformity correction. It provides the possibility of gradual correction of deformities and limb lengthening. Furthermore, knee joint fusion using the Ilizarov technique is considered an ideal treatment for end-stage knee tuberculosis and other infectious arthritides.^[17]^ Despite the high demands of Ilizarov technology, it holds distinct advantages in limb preservation and reconstruction. It enables gradual correction of complex knee deformities while simultaneously lengthening the limb and correcting associated foot deformities. Altaf suggests^[18]^ that, when addressing deformities around the knee joint, gradual distraction through Ilizarov following osteotomy – whether or not accompanied by additional limb lengthening – yields excellent results.

However, the challenges of the Ilizarov technique should not be underestimated. These include the proper construction and manipulation of the Ilizarov frame, the high patient compliance required, the prolonged treatment period, and complications related to the external fixator, such as pin tract infections, discomfort, and aesthetic concerns. In summary, selecting an appropriate and individualized treatment strategy requires a comprehensive assessment of the patient’s specific condition, treatment goals, and the surgeon’s expertise. While the Ilizarov technique presents certain challenges, it provides an effective method for limb preservation and optimizing walking ability in cases of severe knee flexion deformity and shortening.

In the treatment of severe adult knee deformities caused by childhood bone and joint infections, preoperative systematic evaluation and surgical planning are crucial. The patient’s primary goal is to restore function to the affected lower limb, enabling knee extension and achieving near-equal limb length to support bilateral weight-bearing walking. This requires the surgeon to carefully consider how to progressively meet these complex demands in the surgical design. First, based on the patient’s specific condition, such as a flexion deformity exceeding 130° and posterior scar contracture, a staged surgical strategy was formulated. The first stage of the surgery focused on correcting the knee flexion deformity and restoring most of the limb length. The success of this stage was crucial for the patient’s recovery of basic walking ability. The Ilizarov technique effectively controls the movement, correction, and lengthening of bone segments, but it requires technical expertise from the surgeon and strict adherence to fundamental principles. The Ilizarov distraction osteogenesis theory emphasizes that, under the condition of stable external fixator installation, slow, continuous, and stable distraction of the recently osteotomized bone segments allows for the formation of new bone in the gradually widening gap between the bones.^[7]^

The second stage of surgery focused on further lengthening the shortened limb to achieve equal limb lengths. The primary challenge of this stage was the risk of delayed bone healing and potential nonunion, which required a thorough preoperative assessment of bone healing potential, as well as continuous monitoring and appropriate adjustments postoperatively. Moreover, the surgical plan had to take into account the management of scar tissue and the potential risk of infection.

In the second stage of surgery, the primary challenge was addressing the 16 cm shortening of the patient’s affected lower limb. To achieve limb length balance, we chose to simultaneously perform osteotomies in the distal femur and proximal tibia, using a ring external fixator to gradually lengthen the limbs in parallel. During this process, the patient underwent a total of 12 cm of regenerative lengthening, demonstrating the impressive capabilities of the Ilizarov technique in achieving significant limb lengthening. Ghaly et al^[19]^ used the Ilizarov fixator to lengthen both femora and tibiae in children with severe limb length discrepancies caused by fibular hemimelia. The total lengthening achieved was 9.8 cm (range 6–13 cm). Thaller et al^[20]^ conducted a preliminary study on the use of fully implanted magnetic-driven PHENIX nails for limb lengthening in 10 patients with limb shortening, aged 15 to 40 years, with an average lengthening of 4.6 cm (range 1.3–7.6 cm). Three patients required revision surgery due to premature cessation of distraction.

The patient’s femur and tibia were exceptionally slender, making the process of long-segment bone lengthening inherently slow, with a higher likelihood of delayed bone healing. In this case, due to insufficient bone healing at the tibial lengthening site, we performed a stimulation procedure using steel pins at the bone lengthening site to promote bone healing. This is an adjunctive technique used to stimulate bone regeneration. By drilling Kirschner wires into the poorly healed bone region, fresh bone surfaces are created, which in turn stimulate local bone healing.

In complex orthopedic surgeries, the patient’s positive mindset and the physician’s coordinated efforts are crucial for treatment success. A positive patient is better prepared to face the challenges of surgery and recovery, while the physician’s support and encouragement provide emotional and psychological security. With thorough communication, the doctor not only explains the treatment plan but also offers necessary encouragement and guidance throughout the process, helping the patient maintain a positive attitude. This good doctor–patient relationship plays a key role in improving treatment adherence and final recovery outcomes, particularly in complex treatments like those involving the Ilizarov external fixator, which require long-term treatment and high patient cooperation. Adequate communication with the patient and family members is also essential to ensure they have a full understanding of the surgical process, potential risks, and expected outcomes.

## 4. Conclusion

This case demonstrates the effectiveness of the Ilizarov technique in correcting severe deformities and achieving significant limb lengthening in a complex condition caused by childhood osteoarticular infection. The successful outcome was attributed to systematic preoperative evaluation, meticulous surgical planning, and strong patient cooperation. Furthermore, pediatric osteoarticular infections, if not promptly and effectively treated, necessitate careful monitoring for potential limb deformities during subsequent growth and development.

## Author contributions

**Conceptualization:** Ji Wu, Sihe Qin.

**Data curation:** Ji Wu, Sihe Qin.

**Supervision:** Sihe Qin.

**Visualization:** Ji Wu, Sihe Qin.

**Writing – original draft:** Ji Wu.

**Writing – review & editing:** Sihe Qin.

## References

[1] Swarup I Meza BC Weltsch D Jina AA Lawrence JT Baldwin KD. Septic arthritis of the knee in children: a critical analysis review. JBJS Rev. 2020;8:e0069. 32105243 10.2106/JBJS.RVW.19.00069

[2] Darraj H Hakami KM Zogel B Maghrabi R Khired Z. Septic arthritis of the knee in children. Cureus. 2023;15:e45659. 37868524 10.7759/cureus.45659 PMC10590147

[3] Hunter S Chan H Baker JF. Global epidemiology of childhood bone and joint infection: a systematic review. Infection. 2022;50:329–41. 35048321 10.1007/s15010-021-01741-3

[4] Insall JN Dorr LD Scott RD Scott WN. Rationale of the Knee Society clinical rating system. Clin Orthop Relat Res. 1989;248 13–4. 2805470

[5] Ilharreborde B. Sequelae of pediatric osteoarticular infection. Orthop Traumatol Surg Res. 2015;101(1 Suppl):S129–37. 25553604 10.1016/j.otsr.2014.07.029

[6] Agarwal A Rastogi P. Septic sequelae of hip in children: long-term clinicoradiological outcome study. J Pediatr Orthop B. 2021;30:563–71. 33136797 10.1097/BPB.0000000000000828

[7] Gaurav K Vilas J. A new approach to the management of fixed flexion deformity of the knee using Ilizarov’s principle of distraction histogenesis: a preliminary communication. Int J Low Extrem Wounds. 2010;9:70–3. 20483805 10.1177/1534734610371559

[8] Leveille LA Razi O Johnston CE. Rebound deformity after growth modulation in patients with coronal plane angular deformities about the knee: who gets it and how much? J Pediatr Orthop. 2019;39:353–8. 31305378 10.1097/BPO.0000000000000935

[9] Ulusaloglu AC Asma A Silva LC Miller F Mackenzie WG Mackenzie WGS. Growth modulation by tension band plate in achondroplasia with varus knee deformity: comparison of gait analysis measurements. J Pediatr Orthop. 2023;43:168–73. 36583511 10.1097/BPO.0000000000002342

[10] McClure PK Alrabai HM Herzenberg JE. Growth modulation for fixed flexion contracture of the knee: a comparison of two techniques. J Pediatr Orthop B. 2021;30:37–42. 32496748 10.1097/BPB.0000000000000755

[11] Masquijo JJ Artigas C de Pablos J. Growth modulation with tension-band plates for the correction of paediatric lower limb angular deformity: current concepts and indications for a rational use. EFORT Open Rev. 2021;6:658–68. 34532073 10.1302/2058-5241.6.200098 PMC8419796

[12] Kim GK Mortazavi SM Parvizi J Purtill JJ. Revision for stiffness following TKA: a predictable procedure? Knee. 2012;19:332–4. 21839638 10.1016/j.knee.2011.06.016

[13] Parvizi J Tarity TD Steinbeck MJ. Management of stiffness following total knee arthroplasty. J Bone Joint Surg Am. 2006;88(Suppl 4):175–81. 17142446 10.2106/JBJS.F.00608

[14] Gonzalez Della Valle A Leali A Haas S. Etiology and surgical interventions for stiff total knee replacements. HSS J. 2007;3:182–9. 18751792 10.1007/s11420-007-9053-4 PMC2504257

[15] Agarwal S Chakrabarti D Kong K Mayo I Morgan-Jones R. Results of revision knee replacement for patients with isolated fixed flexion deformity after primary or revision knee replacement. Knee. 2021;33:260–5. 34739957 10.1016/j.knee.2021.10.002

[16] Schober TL Abrahamsen C. Patient perspectives on major lower limb amputation – a qualitative systematic review. Int J Orthop Trauma Nurs. 2022;46:100958. 35930959 10.1016/j.ijotn.2022.100958

[17] Sun J Li Q Gao F Xiang Z Huang Q Li L. Application of the Ilizarov technique for knee joint arthrodesis as a treatment for end-stage tuberculosis of the knee. BMC Musculoskelet Disord. 2020;21:579. 32847561 10.1186/s12891-020-03603-9 PMC7447600

[18] Kawoosa AA Wani IH Dar FA Sultan A Qazi M Halwai MA. Deformity correction about knee with Ilizarov technique: accuracy of correction and effectiveness of gradual distraction after conventional straight cut osteotomy. Ortop Traumatol Rehabil. 2015;17:587–92. 27053390 10.5604/15093492.1193011

[19] Ghaly HM El-Rosasy MA Marei AE El-Gebaly OA. Simultaneous femoral and tibial lengthening for severe limb length discrepancy in fibular hemimelia. J Orthop Surg Res. 2023;18:844. 37936235 10.1186/s13018-023-04229-y PMC10631071

[20] Thaller PH Furmetz J Wolf F Eilers T Mutschler W. Limb lengthening with fully implantable magnetically actuated mechanical nails (PHENIX((R>-preliminary results. Injury. 2014;45(Suppl 1):S60–5. 24321414 10.1016/j.injury.2013.10.029

